# Novel ZnO-binding peptides obtained by the screening of a phage display peptide library

**DOI:** 10.1007/s11051-012-1218-5

**Published:** 2012-10-04

**Authors:** Piotr Golec, Joanna Karczewska-Golec, Marcin Łoś, Grzegorz Węgrzyn

**Affiliations:** 1 Laboratory of Molecular Biology (affiliated with the University of Gdańsk), Institute of Biochemistry and Biophysics, Polish Academy of Sciences, Wita Stwosza 59, 80-308 Gdańsk, Poland; 2 Laboratory of Molecular Bacteriology, Intercollegiate Faculty of Biotechnology, University of Gdańsk and Medical University of Gdańsk, Dębinki 1, 80-211 Gdańsk, Poland; 3 Department of Molecular Biology, University of Gdańsk, Wita Stwosza 59, 80-308 Gdańsk, Poland; 4 Institute of Physical Chemistry Polish Academy of Sciences, Kasprzaka 44/52, 01-224 Warsaw, Poland; 5 Phage Consultants, Partyzantów 10/18, 80-254 Gdańsk, Poland

**Keywords:** Zinc oxide, ZnO nanoparticles, ZnO-binding peptides, Phage display

## Abstract

Zinc oxide (ZnO) is a semiconductor compound with a potential for wide use in various applications, including biomaterials and biosensors, particularly as nanoparticles (the size range of ZnO nanoparticles is from 2 to 100 nm, with an average of about 35 nm). Here, we report isolation of novel ZnO-binding peptides, by screening of a phage display library. Interestingly, amino acid sequences of the ZnO-binding peptides reported in this paper and those described previously are significantly different. This suggests that there is a high variability in sequences of peptides which can bind particular inorganic molecules, indicating that different approaches may lead to discovery of different peptides of generally the same activity (e.g., binding of ZnO) but having various detailed properties, perhaps crucial under specific conditions of different applications.

## Introduction

Zinc oxide (ZnO) is a compound widely used in many applications, including construction of solar cells, luminescent materials, and acoustic devices (Goyal et al. 1992; Ezhilvalavan and Kutty 1997; Yu et al. 2005; Shinde et al. 2007). Interestingly, employment of ZnO in biosensors has also been proposed (Gerstel et al. 2006; Park et al. 2009; Tomczak et al. 2009). The possibility to obtain ZnO nanoparticles (i.e., the structures whose at least one dimension is less than 100 nm) significantly broads its practical usefulness (Fan and Lu 2005). It appears that ZnO nanoparticles (which are usually in the size range between 2 and 100 nm, with an average of about 35 nm; Meulenkamp 1998) may be of special interest in biotechnology and nanotechnology (Umetsu et al. 2005; Okochi et al. 2010). In fact, ZnO nanomaterials have been used in sensors, field emission devices, photodetectors, and optical switches (for a review, see Weintraub et al. 2010). Medical applications of ZnO nanoparticles are facilitated due to consideration of ZnO as a safe material, and include anticancer agents, antimicrobial factors, and drug delivery systems (discussed by Li et al. 2008 and Rasmussen et al. 2010). Since ZnO nanoparticles can absorb UV light, they are used in cosmetics, particularly in face or body sunscreen creams (Rasmussen et al. 2010).

The wide use of ZnO nanoparticles stimulated the search for compounds that may specifically bind such structures. In fact, agents that can bind inorganic compounds may be used to build materials with nanoscale precision. Peptides and proteins were demonstrated to be particularly useful in this approach, even when applied to substances not commonly found in biological systems (Brown et al. 2000). Therefore, knowing an extremely high variability of properties of peptides and proteins, and a potential possibility to obtain a peptide that might bind any small molecule of interest, it is reasonable to search for peptides specifically interacting with certain compounds (Seker and Demir 2011). One of the most effective methods in such a search, especially if a target material forms nanoparticles, is screening of peptide libraries (Seker and Demir 2011).

Phage display technique is a method which allows production of various peptides attached to the surface proteins of bacteriophage virions (Castel et al. 2011). This is achieved by genetic engineering of the bacteriophage genome, particularly by fusing genes coding for phage structural proteins with an appropriate DNA fragment, coding for the desired protein or peptide. This may concern either a specific peptide or protein of known functions or a putative one. In the latter case, DNA fragments of randomized sequences can be cloned, and their expression leads to formation of a peptide library, expressed on the bacteriophage surface. Derivatives of phage M13 are among the most widely used vectors in phage display systems (Georgieva and Konthur 2011).

Previously, several ZnO-binding peptides have been reported, which were isolated on the basis of various approaches, including screening of peptide libraries expressed in phage display systems (Kjærgaard et al. 2000; Umetsu et al. 2005; Okochi et al. 2010; Vreuls et al. 2010). Amino acid sequences of these peptides are listed in Table 1. We wanted to probe whether amino acid sequences of ZnO-binding peptides are strictly determined (i.e., the number of possible kinds of ZnO-binding peptides of various sequences is strictly limited) or whether there are many possibilities to form such peptides, whose sequences are significantly different. If the former alternative is true, any new attempts to obtain ZnO-binding peptides should result in obtaining results similar to those published to date. On the other hand, if the latter option is correct, new experiments, differing even slightly in details, should result in isolating peptides which bind ZnO but have amino acid sequences significantly different from those already reported.

**Table 1:** Sequences of ZnO-binding peptides identified in this report and in previously published ones

Peptide name	Amino acid sequence	pI^a^	Remarks	Reference
PG-2	TMGANLGLESPE	3.79	Affinity of PG-2 to ZnO is at least 10 times lower than those of PG-7, PG-8, PG-10, PG-12, PG-14 and PG-17	This report
PG-7	TMGANLGLKWPV	8.41	The consensus sequence determined in this report	This report
ZnO-1	EAHVMHKVAPRP	8.86	Affinity of ZnO-1 to ZnO is at the same level as that of PG-7	(Umetsu et al. 2005)
ZnO-2	QNTATAVSRLSP	9.75	Affinity of ZnO-2 to ZnO is at least 100 times lower than that of ZnO-1	(Umetsu et al. 2005)
ZnO-3	ATHTNQTHALYR	8.80	Affinity of ZnO-3 to ZnO is at least 100 times lower than that of ZnO-1	(Umetsu et al. 2005)
ZnO-4	VSNHKALDYPTR	8.57	Affinity of ZnO-4 to ZnO is at least 100 times lower than that of ZnO-1	(Umetsu et al. 2005)
ZnO-5	DSGRYSMTNHYS	6.74	Affinity of ZnO-5 to ZnO is at least 100 times lower than that of ZnO-1	(Umetsu et al. 2005)
ZnO Okochi-1^b^	HVNLHS	6.92	Affinity of ZnO Okochi-1 to ZnO is at the same level as that of ZnO-1	(Okochi et al. 2010)
ZnO Okochi-2^b^	RCARRY	10.76	Affinity of ZnO Okochi-2 to ZnO is at the same level as that of ZnO-1	(Okochi et al. 2010)
ZnO Okochi-3^b^	HYQSNW	6.74	Affinity of ZnO Okochi-3 to ZnO is at the same level as that of ZnO-1	(Okochi et al. 2010)
ZnO Okochi-4^b^	HWFHPR	9.76	Affinity of ZnO Okochi-4 to ZnO is at the same level as that of ZnO-1	(Okochi et al. 2010)
ZnO-binding peptide^b^	VRTRDDARTHRK	11.54	This peptide has been found with the use of the FimH display system	(Kjærgaard et al. 2000; Vreuls et al. 2010)

^a^Isoelectric point had been calculated using pI/mass program at http://ca.expasy.org

^b^Names have been given by authors of this work, as no specific names were used in the original publications

## Materials and methods

### Peptide phage library

In order to isolate novel ZnO-binding peptides, we have employed a peptide phage library that displays a linear 12-mer peptide on the pIII protein of M13KE phage (Ph.D.-12 Phage Display Peptide Library Kit, New England Biolabs, NEB). This system was successfully used previously to find material-specific peptides (Whaley et al. 2000; Chen et al. 2006; Ahmad et al. 2008).

### Selection and characterization of bacteriophages presenting ZnO-binding peptides

In order to select bacteriophages presenting ZnO-binding peptides on their surfaces, we have utilized synthetic, physically powdered ZnO (Sigma-Aldrich). Panning procedure was performed according to the Phage Display Manual (NEB). Briefly, 10 mg of ZnO powder were washed six times with the TBST buffer (50 mM Tris–HCl pH 7.5, 150 mM NaCl, 0.1 % Tween-20) to remove any ZnO particles that do not sediment during centrifugation at 4,000 × *g* for 1 min (which would negatively interfere with phage isolation at later steps of the procedure) and to establish the equilibrium of buffer conditions of the mixture (which is crucial for effective binding of phages to any surface). 10 μL of phage library was incubated with ZnO in 1 mL of the TBST buffer (at final ZnO concentration of 10 mg/mL) for 1 h at room temperature (RT). This incubation time was chosen on the basis of results of preliminary experiments, in which shorter incubation was less efficient in isolating ZnO-binding phages, while extending the time over 1 h did not change the results significantly. Unbound phages were then separated from ZnO-binding phages by centrifugation (4,000 × *g*, 1 min, RT) and were subsequently removed by decanting the supernatant. The pellet containing ZnO with bound phages was washed ten times with the TBST buffer. Phages which bound ZnO were eluted with 1 mL of 0.2 M glycine–HCl (pH 2.2) for 10 min, and finally neutralized with 150 μL of 1 M Tris–HCl (pH 9.1). Phages were then multiplied on *Escherichia coli* ER2738 (NEB) according to the NEB protocol and used in the next panning procedure (briefly, for phage multiplication, 0.2 mL of an overnight *E. coli* ER2738 culture were mixed with eluted phages, transferred to 20 mL of fresh LB medium in a 250 mL flask, and incubated with shaking (160 rpm) at 37 °C for 5 h). Three rounds of panning were performed. Following the last panning, the eluted phages were titrated on agar plates supplemented with IPTG/XGal as previously described (Łoś et al. 2008). Phages from individual plaques were amplified as described above, and their DNAs were isolated and purified according to Wilson (1993) and sequenced commercially using the 96 gIII sequencing primer (NEB).

## Results and discussion

Experiments performed as described in Materials and methods led to detection of a huge variability (in the range of a few to several orders of magnitude) in efficiencies of binding to ZnO nanoparticles exhibited by different phage clones, as estimated by determining percentages of bound and unbound virions after 1 h incubaction. In fact, some binding (several percent of bound virions) could be observed even for phages that did not expose any foreign peptides on their capsids (data not shown). This might be explained by the fact that no chemical surface could be truly neutral in its effects on adhesion, thus, even naturally occurring phages may attach, to some extent, to any particles. Nevertheless, we have isolated 20 phage clones which were able to bind ZnO very efficiently, i.e., in which at least 99.99 % virions were able to interact with ZnO sufficiently stably to remain bound after the washing procedures.

**Fig. 1:**
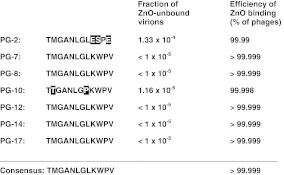
Characterization of selected phage clones which expose ZnO-binding peptides on their virion surfaces. Names of clones and sequences of the exposed peptides are shown, with differences in amino acid residues between peptides marked by* white letters* on the black background. Efficiency of ZnO binding by particular phage clones was estimated by determination of the fraction of virions unbound to ZnO after the washing procedure (see Materials and methods for details)

Of the isolated 20 clones, those revealing the highest affinity to ZnO (clones no. PG-7, PG-8, PG-10, PG-12, PG-14, PG-17) were chosen for more detailed analysis, in which nucleotide sequences of the DNA inserts have been determined. As a semi-negative control, a clone revealing about ten times lower affinity to ZnO (measured as a fraction of virions able to remain bound after the washing procedures), called clone no. PG-2, was also analyzed (Fig. 1). Amino acid sequences of peptides PG-7, PG-8, PG-12, PG-14, and PG-17, exposed on the phage surface, deduced on the basis of determined nucleotide sequences, were identical and red as follows: TMGANLGLKWPV (Fig. 1; Table 1). The amino acid sequence of PG-10 was TTGANLGPKWPV, and that of PG-2 was TMGANLGLESPE. Comparison of the differences between clones binding ZnO relatively strongly (i.e., consisting of less than 1 per 10^5^ virions unbound to ZnO, giving the efficiency of binding >99.999 %; clones PG-7, PG-8, PG-12, PG-14, and PG-17) with those binding ZnO slightly (clone PG-10) or significantly (clone PG-2) weaker indicated that the optimal amino acid sequence of ZnO-binding peptide, isolated under conditions employed in this study, was as follows: TMGANLGLKWPV (Fig. 1). Moreover, it appears that replacement of M with T at position 2 and of L with P at position 8 (as in PG-10) had a minor effect on ZnO binding, while replacement of K with E at position 9, W with S at position 10, and V with E at position 12 (as in PG-2) resulted in a considerable lower affinity of the peptide to ZnO (Fig. 1).

**Fig. 2:**
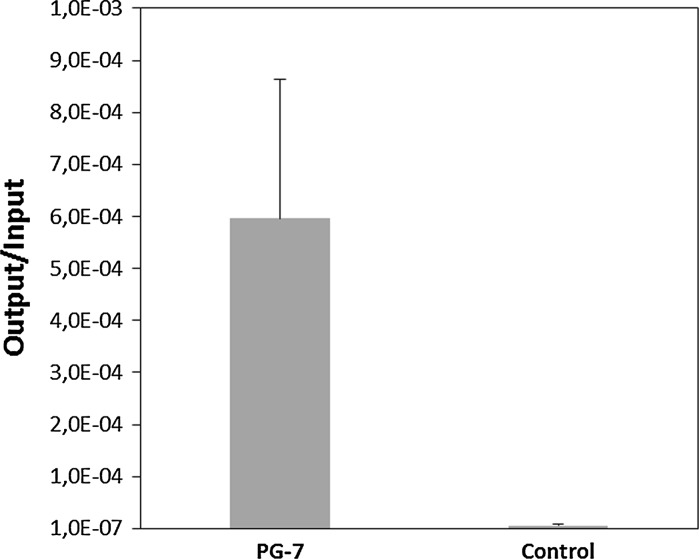
Efficiency of ZnO binding by the phage exposing the PG-7 peptide on the pIII protein of the M13KE phage. In the control experiments, the unmodified M13KE phage was used. The presented results are average values from three experiments with SD represented by *error bars*

The clone no. PG-7 (revealing the optimal ZnO-binding sequence of the phage surface-exposed peptide), has been further characterized. We assessed the efficiency of ZnO binding according to the panning procedure (Sano and Shiba 2003). Binding efficiency was expressed as the ratio of the output phage number (phages eluted, O) to the input phage number (phages incubated with ZnO, I), called the output/input (O/I) ratio. As a control, we employed the M13KE phage with the wild-type pIII protein (NEB). The results of such experiments, presented in Fig. 2, confirmed an efficient binding of the selected peptide to ZnO. This efficiency appears to be similar to those described previously for other peptides isolated as agents that bind ZnO effectively (Kjærgaard et al. 2000; Umetsu et al. 2005; Okochi et al. 2010; Vreuls et al. 2010).

The results of experiments presented in this report indicate that by changing either experimental conditions or approach, it is possible to isolate ZnO-binding peptides of very different amino acid sequences (compare sequences of ZnO-binding peptides presented in Table 1). Therefore, it appears that variability of peptide structures that are able to bind this compound is very high. This implies potential possibilities of searching for ZnO-binding peptides revealing various properties under different reaction conditions. In this light, it is worth reminding that different applications of ZnO may require binding of this compound by peptides under various conditions of temperature, pH, ionic strength, and others (Umetsu et al. 2005).

Finally, one should note that searching for factors that can bind certain compounds, particularly when using the method of phage display-based screening of peptide libraries, is devoted to bio- and bionano-technological applications, and in no way represents processes that occur in nature. The phage display tests demonstrate a huge biological potential of organisms rather than mimic actual biological selection, at least under conditions currently occurring in natural habitats.
